# Harmonizing the diagnosis of citrate accumulation - a call for standardized criteria

**DOI:** 10.1186/s13054-026-06347-5

**Published:** 2026-09-23

**Authors:** Swee Ping Teh, Frank Bidar, Mattia Müller, Beatrice Brunoni, Dmytro Khadzhynov, Manish Kaushik, David Legouis, Sascha David, Thomas Rimmelé, Antoine Schneider

**Affiliations:** 1 Adult Intensive Care Unit, Centre Hospitalier Universitaire Vaudois (CHUV), Lausanne, 1011 Switzerland; 2 Department of Renal Medicine, Sengkang General Hospital, Singapore, Singapore; 3 Department of Anesthesiology and Intensive Care Medicine, University Hospital Louis Pradel, Hospices Civils de Lyon, Bron, France; 4 CarMeN Laboratory, INSERM UMR 1060, Claude Bernard Lyon 1 University, Lyon, France; 5 Institute of Intensive Care Medicine, University Hospital Zurich and University of Zurich, Zurich, Switzerland; 6 Department of Medicine and Surgery, University of Milan-Bicocca, Monza, Italy; 7 KfH Medical Care Centre Berlin‑Mitte, Berlin, Germany; 8 Department of Internal Medicine, St Hedwig Hospital Alexianer, Berlin, Germany; 9 Department of Renal Medicine, Singapore General Hospital, Singapore, Singapore; 10 Division of Intensive Care, University Hospital of Geneva, Geneva, Switzerland; 11 Department of Nephrology, Hannover Medical School, Hannover, Germany; 12 Equipe CARRES, UR2CA, Université Côte d’Azur - CHU de Nice, Nice, France; 13 Medical-Surgical Intensive Care Unit, Pasteur Hospital, CHU de Nice, France; 14 Faculty of Biology and Medicine, University of Lausanne, Lausanne, Switzerland

**Keywords:** Citrate accumulation, Regional citrate anticoagulation, Continuous renal replacement therapy

## Abstract

Regional citrate anticoagulation (RCA) has emerged as the gold-standard strategy for continuous renal replacement therapy (CRRT), yet its widespread adoption remains hindered by perceived complexity, heterogeneous protocols, unclear contraindications, and inconsistent terminology. A central challenge lies in the recognition and management of citrate accumulation, for which no single, reliable biomarker exists. The total-to-ionized calcium (*t*Ca/*i*Ca) ratio is widely utilized as a sensitive surrogate, but its specificity is limited by multiple confounding factors, including serum albumin concentration, acid-base status, and exogenous calcium administration. Current evidence supports a paradigm shift from reliance on static thresholds toward a dynamic, physiology-based assessment to define citrate accumulation. The kinetics of the *t*Ca/*i*Ca ratio, when interpreted alongside evolving metabolic parameters, particularly lactate trends, systemic ionized calcium, and acid-base status, provide a more robust framework for identifying citrate accumulation. Crucially, an elevated *t*Ca/*i*Ca ratio should not indiscriminately prompt the discontinuation of RCA. Targeted adjustments to the CRRT prescription can often safely mitigate citrate burden while preserving circuit patency. In the absence of a validated and widely available method for the direct measurement of plasma citrate levels, we propose a stepwise approach incorporating routinely measured screening tools and confirmatory parameters. Studies incorporating direct plasma citrate concentration measurements should be conducted to validate these criteria alongside readily available bedside surrogate markers. Refining this framework is essential to enhance the safe implementation of RCA and ultimately improve outcomes in critically ill patients.

## Background

High-quality evidence has firmly established the superiority of regional citrate anticoagulation (RCA) over systemic heparin for both (i) maintaining circuit patency and (ii) reducing the risk of bleeding in continuous renal replacement therapy (CRRT) [1, 2]. By significantly prolonging filter lifespan, RCA reduces nursing workload, decreases blood loss, and minimizes filter wastage, thereby lowering the therapy’s carbon footprint, an increasingly vital consideration in sustainable healthcare [3]. Consequently, RCA is recommended as the first-line anticoagulation strategy for all critically ill patients requiring CRRT in the absence of a clinical contraindication [4].

Despite its clear clinical advantages and formal endorsements, the global adoption of RCA remains highly heterogeneous. Data from the STARRT-AKI trial showed that RCA was used in fewer than half of the enrolled patients [5], while a recent survey reported an adoption rate of merely 29% [6]. Persistent barriers to widespread adoption include perceived technical complexity and increased workload, a lack of protocol standardization, regulatory issues, and concerns regarding citrate accumulation.

Citrate accumulation is a complication of RCA that confers a high mortality rate. Although generally considered uncommon, its true incidence remains uncertain, primarily due to the absence of a universally accepted definition. Indeed, citrate is not routinely measurable in clinical laboratories, and the detection of citrate accumulation relies on indirect clinical and biochemical indicators for which no consensus exists. Consequently, the reported incidence of citrate accumulation in the literature shows considerable heterogeneity, ranging from 0.3% to 17% depending on the diagnostic criteria applied [1, 7–9].

In this comprehensive review, we outline the fundamental biochemical principles of RCA with specific emphasis on citrate accumulation. Furthermore, we critically appraise the limitations of current diagnostic definitions and propose a standardized consensus framework to guide clinical practice.

## Principles of regional citrate anticoagulation in CRRT

RCA requires the administration of a citrate (C_6_H_5_O_7_; ~189 Daltons) solution proximally into the extracorporeal circuit. Citrate exerts its anticoagulant effect by chelating ionized calcium (*i*Ca^2+^), an essential cofactor in the coagulation cascade. This ionic binding leads to the formation of soluble calcium-citrate complexes (CCC; ~298 Daltons) [10, 11]. Effective anticoagulation is achieved when the post-filter *i*Ca^2+^ concentration is maintained below 0.35 mmol/L, which typically requires an initial citrate dose of 4 mmol per liter of blood [12]. At this dose, exogenous citrate does not exhibit direct cellular or organ toxicity.

While the sieving coefficient for CCC is approximately 1.0, around 50% of CCC is cleared extracorporeally - a fraction governed by the relationship between effluent flow rate and plasma flow rate. The remaining fraction enters the systemic circulation as the citrate load, and is metabolized within the mitochondria of the liver, kidneys and skeletal muscle through the oxygen-dependent Krebs cycle, also termed the citric acid cycle [13]. This process generates ATP; once citrate is metabolized, the concomitant sodium load raises the plasma strong ion difference, with one mmol of trisodium citrate provides the same buffer as 3 mmol sodium bicarbonate, assuming the citrate is completely metabolized, thereby exerting a net alkalinizing effect [14]. Thus, citrate acts both as an anticoagulant locally and, when adequately metabolized, as a systemic alkalinizing buffer.

As citrate accumulation represents the dynamic balance between systemic delivery and clearance, exposure to CCC can be actively mitigated by decreasing blood flow rate to reduce the citrate load, or by increasing dialysate or post-filter replacement fluid to augment extracorporeal clearance.

To compensate for the calcium lost in the effluent (in the form of both CCC and unbound *i*Ca^2+^), a compensatory calcium infusion is administered via the return line to maintain a systemic *i*Ca^2+^ concentration of 1.10–1.20 mmol/L.

Depending on institutional protocols, CRRT platforms, and fluid availability, RCA can be tailored across diverse operational modalities including continuous veno-venous hemodialysis (CVVHD), hemofiltration (CVVH) or hemodiafiltration (CVVHDF). Table 1 provides an overview of the different types of citrate solutions available and their chemical composition. While calcium-free dialysate and replacement fluids are standard for RCA-CRRT, specific fluid compositions and operational protocols vary across centers and commercial platforms. Generally, these formulations feature reduced sodium and bicarbonate concentrations to offset the inherent risks of systemic hypernatremia and metabolic alkalosis.

**Table 1:** Type and chemical composition of commonly used solutions for regional citrate anticoagulation

Solution type	Solution name	Sodium (mmol/L)	Citrate (mmol/L)	Citric acid (mmol/L)	Glucose (mmol/L)	Chloride (mmol/L)	Potassium (mmol/L)	Hydrogen ion (mmol/L)	Magnesium (mmol/L)	Osm (mOsm/L)	pH
Concentrated solutions	Acid Citrate Dextrose Solution A (ACD-A)	224.4	74.8	38.1	124	N/A	N/A	114	N/A	N/A	N/A
	Trisodium Citrate Anticoagulant (TSC)* (e.g.: Cifoban®)	408	136	N/A	N/A	N/A	N/A	N/A	N/A	544	7.1–7.5
Diluted citrate solutions	Regiocit®	140	18	N/A	N/A	86	N/A	N/A	N/A	244	~7.4
	CitraSol HF-Cit-Pre®	139.9	13.3	N/A	5	104	3	N/A	0.5	266	4.5 - 6.5

Note: Data compiled from official manufacturer specifications

*Values reflect standard 4% trisodium citrate formulations (such as Cifoban®, Fresenius Medical Care, Bad Homburg, Germany)

Abbreviations: Osm – osmolarity, N/A – not available (indicate the absence of the component from the specific solution’s formulation)

## Citrate accumulation

Citrate accumulation occurs when citrate administration outpaces the body’s metabolic capacity. This metabolic mismatch typically arises in conditions that impair mitochondrial oxidative metabolism – most notably profound shock states with compromised tissue oxygen delivery, severe hepatic dysfunction, or a combination of both. Left untreated, the incomplete metabolism of the CCC results in two primary biochemical consequences: severe systemic ionized hypocalcemia, owing to the inadequate release of bound calcium, and progressive metabolic acidosis. This acidosis is driven by the accumulation of unmetabolized citrate in plasma: as an unmeasured anion, it lowers the strong ion difference and widens the anion gap, producing a metabolic acidosis. The confluence of these profound metabolic derangements can ultimately induce myocardial depression and exacerbate circulatory dysfunction.

### Clinical approach

**Fig. 1:**
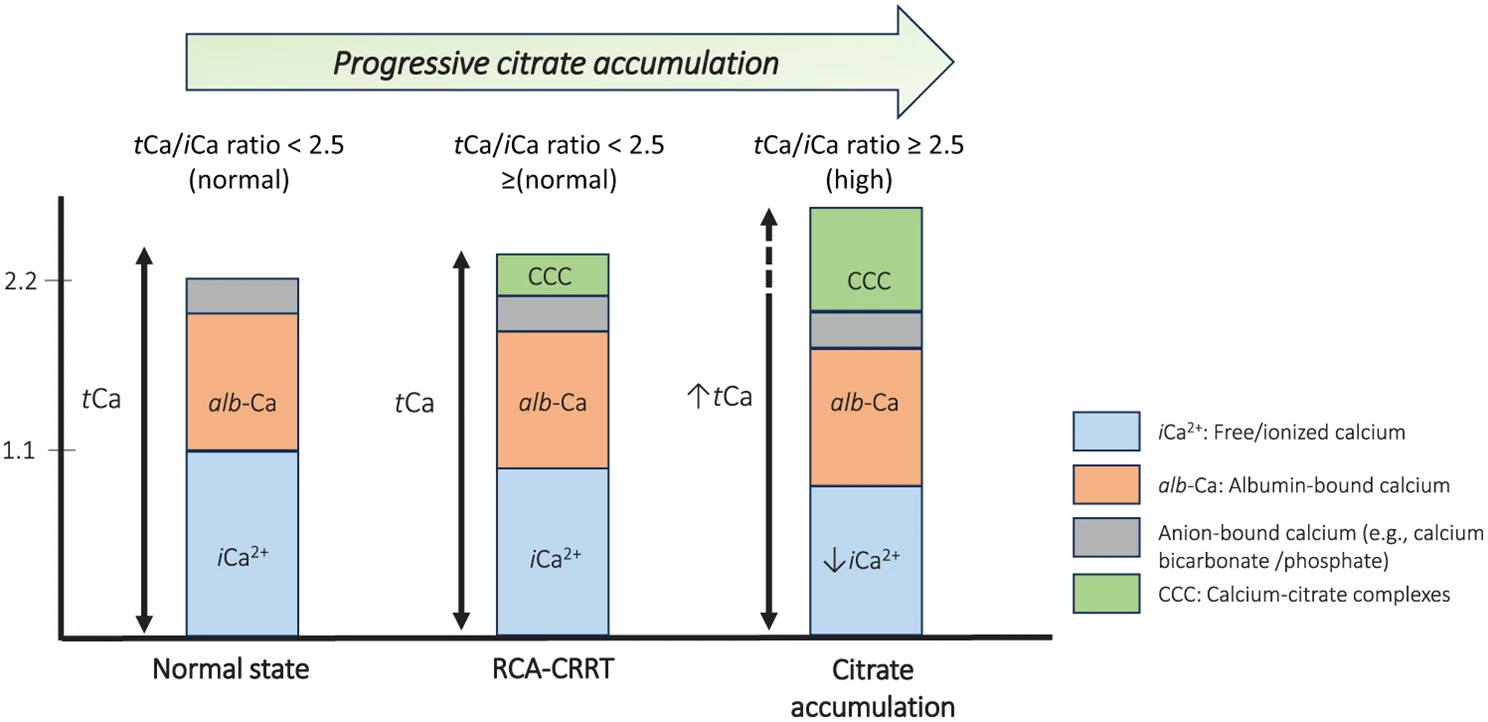
Changes in serum calcium fractions during progressive citrate accumulation in RCA-CRRT. This figure illustrates the distribution of serum calcium fractions across three clinical states: a baseline normal state, uncomplicated RCA-CRRT, and during progressive citrate accumulation. Measured _*t*_Ca/_*i*_Ca represents the fraction occupied by CCC, serving as a surrogate marker of citrate accumulation. Abbreviations: alb-Ca – albumin-bound calcium; CCC – calcium-citrate complexes; *i*Ca^2+^ – free/ionized calcium; *t*Ca – total calcium; _*t*_Ca/_*i*_Ca – total calcium-to-ionized calcium ratio

To date, there is no universally accepted definition for citrate accumulation. Direct quantification of plasma citrate is unavailable in routine clinical laboratories. Current diagnostic approaches rely on the identification of indirect biochemical surrogates. This diagnostic framework typically incorporates a classic triad of parameters (Fig. 1):

- A progressive decline in systemic ionized calcium (*i*Ca^*2+*^), fully or partially compensated by an increase in the calcium substitution rate,
- A new-onset, unexplained worsening of anion-gap metabolic acidosis,
  and
- A progressive or rapid rise in the *total*-to-*ionized* calcium (*t*Ca/*i*Ca) ratio.

In clinical practice, the first two components of this triad are frequently regarded as warning signs, whereas the latter is often considered confirmatory of impaired citrate metabolism. Historically, a *t*Ca/*i*Ca ratio threshold of 2.5, which corresponds to a plasma citrate concentration range of 0.85–1.5 mmol/L, has been proposed to define citrate accumulation [15–17]

### Timing of onset

Current evidence suggests that clinical manifestations of citrate accumulation commonly develop within the first 24 hours following RCA-CRRT initiation [8, 18, 19], although initial uncomplicated RCA-CRRT does not eliminate the possibility of later accumulation, especially in the setting of deterioration of clinical status. Bidar et al. demonstrated that 90% of episodes manifested within this initial 24-hour period, with 57% occurring within the first 6 hours. Patients with immediate-onset accumulation exhibited a substantial baseline metabolic burden, characterized by markedly elevated lactate level (14 mmol/L [IQR 12;15]) and severe metabolic acidosis (pH 7.10 [IQR 7.06; 7.20]) consistent with profound tissue hypoperfusion and compromised systemic metabolism [8]. Crucially, timely adjustment of RCA-CRRT prescription has been shown to facilitate rapid improvement in systemic ionized calcium levels and normalization of the *t*Ca/*i*Ca ratio [8, 19, 20].

## Predictors of citrate accumulation

### Liver failure

Although baseline liver function is important for risk-stratification metrics before initiating RCA, isolated hepatic failure alone does not necessarily preclude its utilization. Data from the Liver Citrate Anticoagulation Threshold (L-CAT) study and subsequent meta-analyses indicate that circuit patency can be safely maintained with rigorous metabolic monitoring. Among patients with severe liver failure, the pooled incidence of citrate accumulation was approximately 12% when defined solely by an elevated *t*Ca/*i*Ca ratio [21–23].

Furthermore, an observational study involving patients with a mean Model for End-stage Liver Disease (MELD) score of 19.2 demonstrated that RCA-CRRT could be successfully continued despite a *t*Ca/*i*Ca ratio exceeding 2.5 (observed in 23.2% of the cohort). This continuity was achieved through increasing dialysate flow to enhance clearance and reducing blood flow to limit total citrate delivery [24].

Biochemically, this underscores that citrate metabolism is not strictly confined to hepatic mitochondria but also involves extra-mitochondrial and extra-hepatic pathways including cytosolic processes linked to the Cori Cycle in skeletal muscle [25]. Consequently, citrate accumulation is typically driven by multiorgan failure rather than isolated hepatic dysfunction.

### Hyperlactatemia

Hyperlactatemia, particularly when coupled with hemodynamic instability, serves as a robust predictor of citrate accumulation. An initial serum lactate > 10 mmol/L is associated with an “immediate” onset (occurring within the first six hours of therapy) of citrate accumulation [8]. However, a single static lactate measurement carries a high negative predictive value but a poor positive predictive value for citrate accumulation. For instance, in the Khadhzynov series, only 14 (6.3%) of the 221 patients with an initial lactate > 4 mmol/L ultimately developed citrate accumulation [18]. Conversely, dynamic lactate kinetics offer superior predictive performance. Specifically, a failure to achieve lactate clearance within 18 hours serves as an early, reliable indicator of impending citrate accumulation [18, 25] In states of profound circulatory shock, severe microcirculatory dysfunction concurrently increases serum lactate and disrupts alternative extra-hepatic metabolic routes, thereby accelerating citrate accumulation [26]. Viewed collectively, citrate accumulation may reflect not merely a treatment-related complication, but a broader state of global “metabolic exhaustion”.

### Other predictors of citrate accumulation

Several drug intoxications, including metformin, propofol, linezolid and tenofovir can induce mitochondrial dysfunction, thereby impairing citrate metabolism [26]. Despite this risk, several studies have established the safety of RCA across renal replacement therapy for metformin-associated lactic acidosis, provided that metformin is adequately cleared [27].

Furthermore, membrane clogging may increase the risk of citrate accumulation by hindering adequate citrate clearance across the filter membrane.

## Controversies regarding citrate accumulation definitions

The absence of a uniform diagnostic definition for citrate accumulation frequently leads to under- or over-reporting of this complication, potentially fostering an unwarranted perception of clinical risk, particularly in centers with limited experience. For instance, while the classic *t*Ca/*i*Ca ratio threshold of 2.5 appears highly sensitive for detecting accumulation, it is very likely to critically lack specificity. The ongoing debate in contemporary literature regarding optimal diagnostic cut-offs for citrate accumulation should be evaluated through the lens of three critical elements: clinical confounders, the impact of hypoalbuminemia, and the relevance of dynamic metabolic trends.

### Confounders of the association between *t*Ca/*i*Ca ratio and citrate accumulation

The *t*Ca/*i*Ca ratio can be influenced by several factors beyond citrate accumulation. These include alterations in albumin concentration, acid-base status, exogenous calcium supplementation and measurement variability. Accordingly, these markers should be interpreted cautiously within the broader clinical and biochemical context [28].

#### Effect of albuminemia

As approximately 40% of extracellular calcium is physiologically bound to albumin, hypoalbuminemia lowers total calcium without affecting ionized calcium (*i*Ca^2+^) [29]. Utilizing an albumin-corrected calcium ratio has been proposed to improve diagnostic sensitivity. Indeed, two recent retrospective cohorts employing the albumin-corrected calcium ratio reported a high citrate accumulation incidence of 17 and 19% [9, 30]. However, a head-to-head comparison of an albumin-corrected calcium ratio versus the conventional *t*Ca/*i*Ca ratio, using measured plasma citrate as the reference, did not show improved performance for detecting citrate accumulation [31].

The reliability of albumin-corrected calcium measurements is uncertain in critically ill populations [32]. Notably, in the study by Meier-Kriesche et al., which established the value of 2.5 as a cutoff to define accumulation, patients with citrate accumulation had a low baseline albumin and no correction was applied (2.4 ± 0.7 g/dL) [33]. Hence, the 2.5 cut-off cannot directly be transposed to an albumin-corrected calcium ratio. A transition to such an adjusted metric will require prospective validation to define an appropriate, optimized cutoff.

### Other variables

Several other factors may independently confound the relationship between the *t*Ca/*i*Ca ratio and citrate accumulation. Certain disease processes (such as hyperparathyroidism and sarcoidosis), exogenous calcium administration, and variations in calcium compensation during CRRT can independently modify total calcium, distorting the ratio without reflecting true citrate burden [34]. In addition, preanalytical and analytical factors, including sampling conditions, delays in measurement, and assay variability for ionized calcium, may affect reliability. Altogether, these elements underscore the need to interpret the ratio considering clinical context and temporal trends rather than as a standalone marker [35].

### Epidemiological disparities and sensitivity of the isolated threshold *t*Ca/*i*Ca ratio

Varying diagnostic stringency further complicates estimates of citrate accumulation incidence. In a large observational study (*n* = 2,080), a liberal, uncorrected *t*Ca/*i*Ca ratio threshold ≥ 2.3 used for screening identified 8.5% of patients on RCA-CRRT as potentially affected [8] However, the application of strict multiparametric criteria [18] halved this incidence, which was further reduced to 3.7% following independent clinical adjudication [8]. Beyond diagnostic criteria, the observation of very high incidences of citrate accumulation in series originating from Asia, raises the possibility of ethnic or genetic variations in citrate metabolism [19].

### Prognostic relevance

The prognostic relevance of the *t*Ca/*i*Ca ratio remains a subject of ongoing debate. Although elevated ratios are often associated with increased mortality, this association typically weakens after multivariable adjustment for lactate, a key marker of tissue hypoperfusion, impaired metabolism, and overall illness severity [9].

It is essential to distinguish between diagnostic thresholds used to identify citrate accumulation and prognostic thresholds associated with clinical outcomes. A threshold optimized for predicting mortality does not necessarily represent the optimal cutoff for diagnosing citrate accumulation [36]. Patients with elevated lactate usually present with a significantly higher baseline severity of illness, characterized by elevated SAPS scores, which independently drive a higher risk of death. Hence, an isolated elevation in the *t*Ca/*i*Ca ratio is merely a surrogate for citrate accumulation and lacks direct, causal transitivity to hard clinical outcomes like mortality. Further research is required to clarify whether the *t*Ca/*i*Ca ratio adds independent prognostic value beyond readily available metabolic markers of shock severity, such as lactate kinetics [37].

## Proposal for a consensus definition

In the absence of a validated and widely available method for the direct measurement of plasma citrate levels, we propose a stepwise approach including routinely measured screening tools and confirmatory parameters. Ideally, future advancements in direct citrate measurement should supersede the need for these surrogate markers.

### Screening strategy

**Fig. 2:**
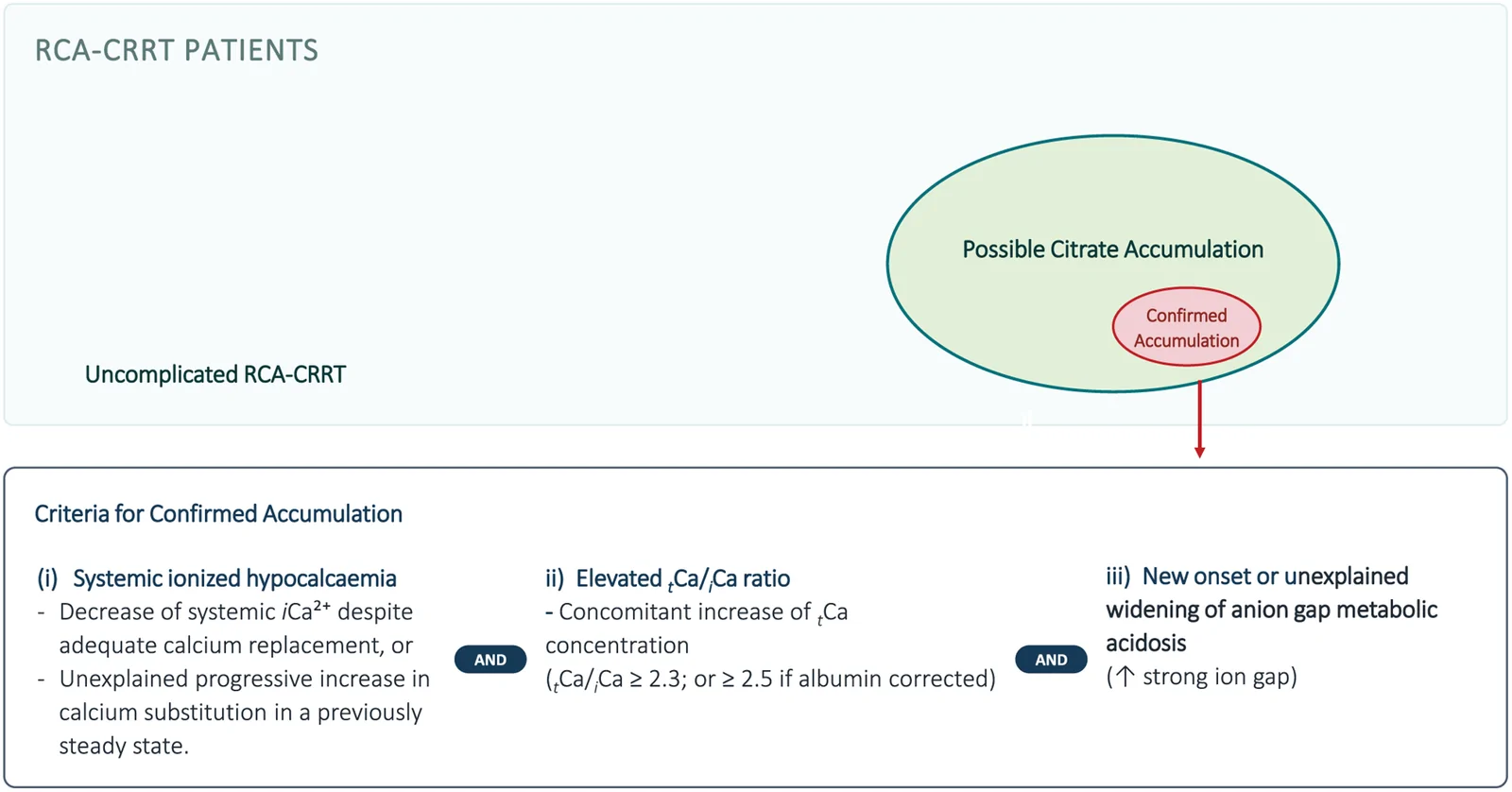
Proposed diagnostic framework for citrate accumulation in patients on RCA-CRRT. The framework distinguishes between possible citrate accumulation and confirmed citrate accumulation based on evolving biochemical trends in a small subset of patients receiving RCA-CRRT. Possible citrate accumulation is suggested by unexplained progressive systemic ionized hypocalcemia or an increasing calcium substitution deviating from a previous steady state, or a progressive increase in the *t*Ca/*i*Ca ratio ≥ 2.3, or a rapid increase in the ratio not meeting the threshold. This threshold serves as an early warning trigger that mandates heightened surveillance rather than immediate treatment cessation. Conversely, confirmed citrate accumulation requires (**i**) systemic ionized hypocalcemia despite adequate calcium replacement, or an unexplained progressive rising calcium substitution from baseline in a previously steady state, and (**ii**) an elevated *t*Ca/*i*Ca ratio ≥ 2.3 (or ≥ 2.5 if albumin corrected), and (**iii**) new or unexplained progressive deterioration of metabolic acidosis – a new onset or widening of anion-gap metabolic acidosis, an increased in strong ion gap, or a parallel downward trajectory in base excess. Abbreviations: *i*Ca^2+^ – ionized calcium; *t*Ca – total calcium; *t*Ca/iCa – total calcium-to-ionized calcium ratio; RCA-CRRT – regional citrate anticoagulation continuous renal replacement therapy

We propose the following bedside screening tools (Fig. 2):

i. Unexplained progressive rise in calcium substitution requirement - particularly when it represents a deviation from the patient’s previous steady state and/or a decreased systemic ionized calcium;
ii. An elevated *t*Ca/*i*Ca ratio (≥2.3) or an elevated albumin-corrected calcium ratio;
iii. A progressive or rapid rise in the *t*Ca/*i*Ca ratio not meeting the threshold.

### Confirmed citrate accumulation

i. Systemic ionized hypocalcemia: a decrease of systemic *i*Ca^2+^ despite adequate calcium replacement, or unexplained progressive increase in calcium substitution in a previously steady state; AND
ii. Elevated *t*Ca/*i*Ca ratio: Concomitant increase in *t*Ca (*t*Ca/*i*Ca ≥ 2.3, or ≥2.5 if albumin corrected); AND
iii. New or unexplained deterioration of metabolic acidosis: a progressive or new onset widening of anion-gap metabolic acidosis, an increased strong ion gap, or a parallel downward trajectory in base excess.

To ensure clinical accuracy, the same metric must be consistently used throughout the monitoring period. The presence of any of these parameters should prompt a medical evaluation to determine the need for therapy adjustment or treatment interruption. These tools should be used to support clinical decision-making and should not be interpreted as a standalone diagnostic criterion or as a definitive trigger to discontinue RCA (see below).

## Citrate accumulation management

Clinical evaluation and integration of the clinical context are key to the risk assessment of possible citrate accumulation. The situation is then stratified into three categories (i) unlikely citrate accumulation (ii) possible citrate accumulation, and (iii) confirmed citrate accumulation.

### Clinical evaluation

**Fig. 3:**
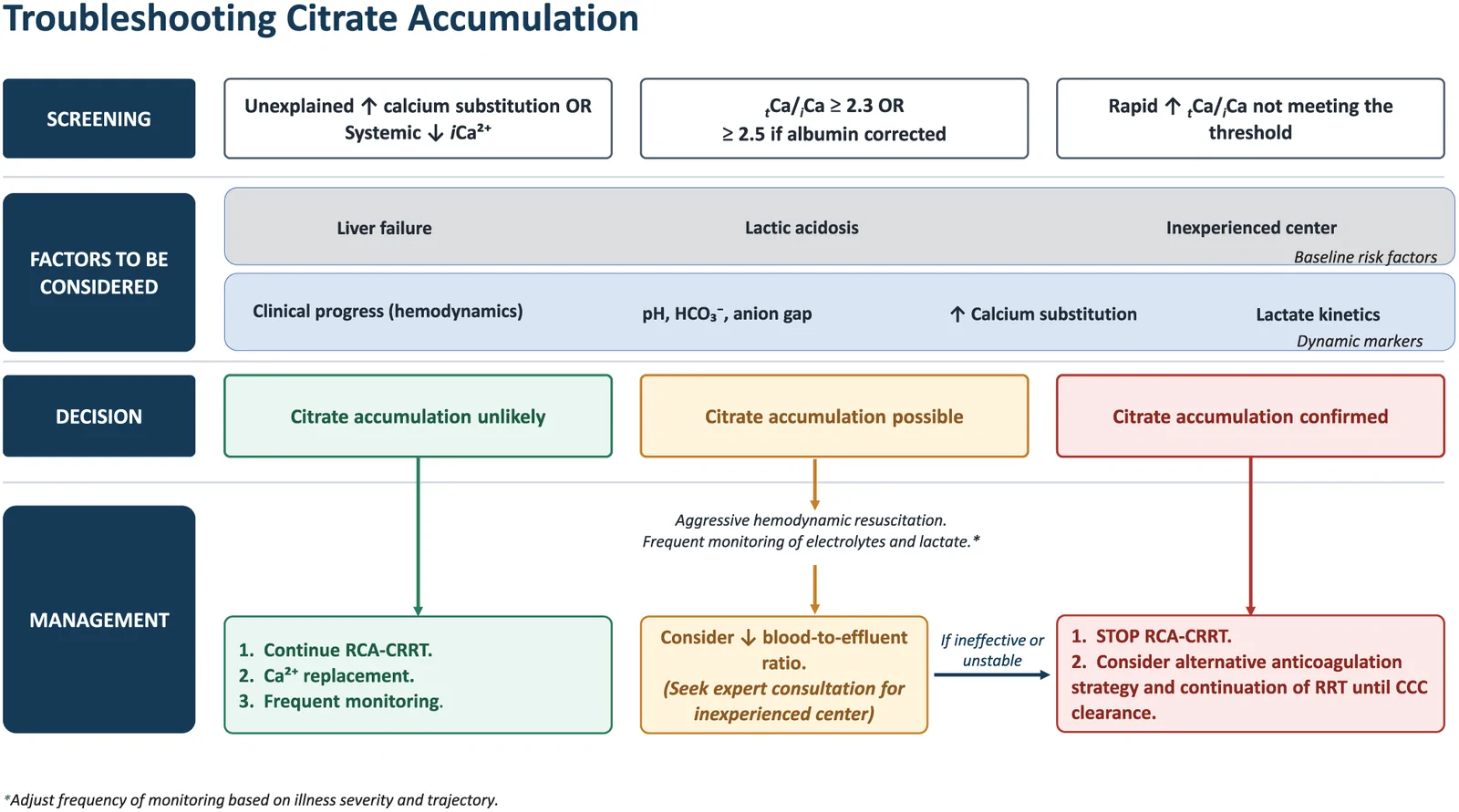
Algorithm for detection and management of citrate accumulation during RCA-CRRT. This structured algorithm outlines the pathway for managing potential citrate accumulation across four sequential phases: screening, evaluation of factors to be considered, decision-making, and management. It begins with screening based on systemic *i*Ca^2+^ levels, calcium substitution requirements, and _*t*_Ca/_*i*_Ca ratio. Identified risks prompt an evaluation phase with baseline risk factors (such as liver failure, lactic acidosis, and center experience) and dynamic markers (including clinical hemodynamic, acid-base status like pH, HCO_3_^-^, calcium substitution rate and lactate kinetics) to determine the likelihood of accumulation. In “possible citrate accumulation,” RCA can be continued with a reduced blood-to-effluent ratio (seeking expert consultation if the center is inexperienced), coupled with intensified monitoring of electrolytes and lactate. If these interventions fail, or if citrate accumulation is confirmed, RCA-CRRT should be discontinued in favor of an alternative anticoagulation technique, with dialysis continued to facilitate the clearance of CCC. Abbreviations: Ca^2+^ – Calcium; CCC – calcium-citrate complexes; HCO_3_^-^ – Bicarbonate; *i*Ca^2+^ – ionized calcium; *t*Ca – total calcium; *t*Ca/*i*Ca – total calcium-to-ionized calcium ratio; RCA – regional citrate anticoagulation

Clinical evaluation triggered by positive screening criteria should integrate numerous parameters (Fig. 3) among which the clinical trajectory, lactate kinetics, the dynamics of the *t*Ca/*i*Ca ratio are key.

Lactate trends help distinguish citrate accumulation from other causes of high anion gap acidosis while acknowledging frequent overlap with shock states. Worsening hemodynamic instability or rising vasopressor needs both raise pretest probability and identify a resuscitation target. A rapid rise in the *t*Ca/*i*Ca ratio or calcium requirement is more concerning than a stable pattern. Finally, the underlying liver function should be considered given the disproportionate risk of accumulation in patients with impaired citrate clearance capacity [38].

At the end of this evaluation, patients should be stratified into one of the following three categories: (i) citrate accumulation unlikely; (ii) citrate accumulation possible; or (iii) citrate accumulation confirmed.

### Citrate accumulation unlikely

When comprehensive evaluation suggests that citrate accumulation unlikely, standard RCA-CRRT with conventional monitoring may be safely continued. Isolated systemic hypocalcemia can be easily managed by intravenous calcium administration alongside frequent monitoring of systemic *i*Ca^2+^.

As an initially uncomplicated course with RCA does not guarantee continued tolerance if the patient’s clinical condition subsequently deteriorates, clinical vigilance must remain ongoing, as subsequent alterations in tissue perfusion or organ function can alter the balance between citrate delivery and systemic metabolism at any point during therapy.

### Possible citrate accumulation

Citrate accumulation is considered as possible in the presence of positive screening criteria (escalating demand for calcium substitution or a progressive rise in the *t*Ca/*i*Ca ratio or albumin-corrected calcium ratio, whether meeting the threshold or not). In this situation, RCA can be continued, provided electrolyte and pH monitoring is intensified and the patient undergoes regular clinical assessment. Particular attention should be paid to the anion gap and lactate level.

In most cases, this can be mitigated by adjusting the prescription to lower the blood-to-effluent ratio, either by decreasing the blood flow rate or increasing the dialysate or post-filter replacement fluid flow rates. These adjustments reduce citrate delivery to the patient, aiming to restore a manageable citrate balance and limit further accumulation while preserving the benefits of regional anticoagulation [7, 19, 24].

If these interventions fail to improve biochemical markers, clinicians should consider transitioning to an alternative anticoagulation strategy (see below), while maintaining dialysis to enable continued clearance of CCC.

### Confirmed citrate accumulation

Confirmed citrate accumulation is rare but carries a very high mortality. RCA should be interrupted immediately, and an alternative anticoagulation strategy for CRRT should be considered for the continuation of renal replacement therapy until the clearance of CCC which typically takes at least 12 hours [20] and the resolution of clinical or biochemical manifestations, provided an independent indication for RRT remains.

In this situation, the primary clinical focus should shift to restoring tissue perfusion and correcting the underlying shock state in order to restore mitochondrial citrate metabolism. Indeed, confirmed citrate accumulation should be regarded not merely as a circuit-related complication, but as a marker of severe underlying illness, typically profound shock with impaired hepatic and/or muscular citrate metabolism.

## Future recommendation for research

Future studies investigating citrate-related complications should prioritize standardization by adopting our proposed stepwise approach, which includes (i) standardized criteria to identify patients at risk of citrate accumulation, incorporating both the *t*Ca/*i*Ca ratio and the albumin-corrected calcium ratio, and (ii) consistent reporting of confirmatory parameters.

Although these approaches may be difficult to implement in retrospective studies leveraging large datasets, they are necessary to overcome the limitations of “one-size-fits-all” criteria, which risk overestimating the true incidence of citrate accumulation.

Finally, studies incorporating direct plasma citrate concentration measurements should be conducted to validate diagnostic criteria alongside routinely available bedside surrogate markers.

## Conclusions

A unified nomenclature and a standardized diagnostic framework are needed. Shifting from static thresholds to a dynamic, integrative assessment of metabolic kinetics with a stepwise approach may reduce diagnostic ambiguity and improve real-world outcomes for critically ill patients.

## Data Availability

No datasets were generated or analysed during the current study.

## Abbreviations

ACD-A: Acid Citrate Dextrose Solution A
Alb: Albumin
ATP: Adenosine triphosphate
CCC: Calcium-citrate complexes
CRRT: Continuous renal replacement therapy
CVVH: Continuous veno-venous hemofiltration
CVVHD: Continuous veno-venous hemodialysis
CVVHDF: Continuous veno-venous hemodiafiltration
HCO_3_^-^: Bicarbonate
*i*Ca^2+^: Ionized calcium
L-CAT: Liver Citrate Anticoagulation Threshold
MELD: Model for End-stage Liver Disease
RCA: Regional citrate anticoagulation
SAPS: Simplified Acute Physiology Score
*t*Ca: Total calcium
*t*Ca/*i*Ca: Total-to-ionized calcium ratio
TSC: Trisodium Citrate Anticoagulant

## References

[1] Zarbock A, Kullmar M, Kindgen-Milles D, Wempe C, Gerss J, Brandenburger T, et al. Effect of regional citrate anticoagulation vs systemic heparin anticoagulation during continuous kidney replacement therapy on dialysis filter life span and mortality among critically ill patients with acute kidney injury: a randomized clinical trial. JAMA. 2020;324(16):1629–39. 10.1001/jama.2020.18618. PMC7585036 33095849

[2] Zhou Z, Liu C, Yang Y, Wang F, Zhang L, Fu P. Anticoagulation options for continuous renal replacement therapy in critically ill patients: a systematic review and network meta-analysis of randomized controlled trials. Crit Care. 2023;27(1):222. 10.1186/s13054-023-04519-1. PMC10249230 37287084

[3] De Simone EA-O, Guarino A, Pozzato M, Roccatello DA-O, Sciascia SA-O, Fenoglio RA-O. Regional citrate anticoagulation in CRRT: successes, pitfalls, and sustainability from a long-term single-center experience. J Clin Med. 2026, Feb, 27;15(5):1807. 10.3390/jcm15051807 PMC12986174 41827223

[4] Group KDIGOKAKIW. KDIGO clinical practice guideline for acute kidney injury. Kidney Int. 2012;2:1–138.

[5] Bagshaw SM, Wald R, Adhikari NKJ, Bellomo R, da Costa BR, Dreyfuss D, et al. Timing of initiation of renal-replacement therapy in acute kidney injury. N Engl J Med. 2020, Jul, 16;383(3):240–51. 10.1056/NEJMoa2000741 32668114

[6] Boldt D, Busse L, Chawla LS, Flannery AH, Khanna A, Neyra JA, et al. Anticoagulation practices for continuous renal replacement therapy: a survey of physicians from the United States. Ren Fail. 2023;45(2):2290932. 10.1080/0886022X.2023.2290932 PMC11001369 38073554

[7] Bachmann D, Monard C, Kelevina T, Ahmad Y, Pruijm M, Chiche JD, et al. Generalization of regional citrate anticoagulation for continuous renal replacement therapy is not associated with an increased rate of severe complications. J Crit Care. 2025, Jun;87:155032. 10.1016/j.jcrc.2025.155032 39954302

[8] Bidar F, Chardon N, Darnajoux Q, Petit M, Fellahi JL, Aubrun F, et al. Clinical and biological profiles associated with the time of occurrence of citrate accumulation in patients receiving continuous renal replacement therapy. Crit Care. 2025;29(1):407. 10.1186/s13054-025-05648-5. PMC12465975 41013710

[9] Muller MM, Weber A, Bartussek J, Bachmann J, Buhlmann A, Kadler G, et al. Incidence, severity, and predictors of citrate accumulation during continuous kidney replacement therapy in the critically ill. Crit Care. 2025;29(1):468. 10.1186/s13054-025-05691-2. PMC12581596 41184940

[10] Legrand M, Tolwani A. Anticoagulation strategies in continuous renal replacement therapy. Semin Dial. 2021;34(6):416–22. 10.1111/sdi.12959. 33684244

[11] Schneider AG, Journois D, Rimmele T. Complications of regional citrate anticoagulation: accumulation or overload? Crit Care. 2017;21(1):281. 10.1186/s13054-017-1880-1. PMC5694623 29151020

[12] Calatzis A, Toepfer M, Schramm W, Spannagl M, Schiffl H. Citrate anticoagulation for extracorporeal circuits: effects on whole blood coagulation activation and clot formation. Nephron. 2001, Oct;89(2):233–36. 10.1159/000046075. 11549910

[13] Davenport A, Tolwani A. Citrate anticoagulation for continuous renal replacement therapy (CRRT) in patients with acute kidney injury admitted to the intensive care unit. NDT Plus. 2009;2(6):439–47. 10.1093/ndtplus/sfp136. PMC4421325 25949376

[14] Oudemans-van Straaten HM, Kellum JA, Bellomo R. Clinical review: anticoagulation for continuous renal replacement therapy-heparin or citrate? Crit Care. 2011;15(1):202. 10.1186/cc9358. PMC3222015 21345279

[15] Kramer L, Bauer E, Joukhadar C, Strobl W, Gendo A, Madl C, et al. Citrate pharmacokinetics and metabolism in cirrhotic and noncirrhotic critically ill patients. Crit Care Med. 2003;31(10):2450–55. 10.1097/01.CCM.0000084871.76568.E6 14530750

[16] Hetzel GR, Taskaya G, Sucker C, Hennersdorf M, Grabensee B, Schmitz M. Citrate plasma levels in patients under regional anticoagulation in continuous venovenous hemofiltration. Am J Kidney Dis. 2006;48(5):806–11. 10.1053/j.ajkd.2006.07.016. 17060000

[17] To HHM, Kwan AMC, Leung NYY, Chan WM, Ngai CW, Wong ASK, et al. A prospective study on serum citrate levels and clinical correlations in patients receiving regional citrate anticoagulation. Clin Kidney J. 2023;16(2):285–92. 10.1093/ckj/sfac223 PMC9900571 36755836

[18] Khadzhynov D, Dahlinger A, Schelter C, Peters H, Kindgen-Milles D, Budde K, et al. Hyperlactatemia, lactate kinetics and prediction of citrate accumulation in critically ill patients undergoing continuous renal replacement therapy with regional citrate anticoagulation. Crit Care Med. 2017;45(9):e941–6. 10.1097/CCM.0000000000002501 28538441

[19] Poh CB, Tan PC, Kam JW, Siau C, Lim NL, Yeon W, et al. Regional citrate anticoagulation for continuous renal replacement therapy - a safe and effective low-dose protocol. Nephrology. 2020;25(4):305–13. 10.1111/nep.13656. 31469465

[20] Khadzhynov D, Schelter C, Lieker I, Mika A, Staeck O, Neumayer HH, et al. Incidence and outcome of metabolic disarrangements consistent with citrate accumulation in critically ill patients undergoing continuous venovenous hemodialysis with regional citrate anticoagulation. J Crit Care. 2014;29(2):265–71. 10.1016/j.jcrc.2013.10.015 24360392

[21] Slowinski T, Morgera S, Joannidis M, Henneberg T, Stocker R, Helset E, et al. Safety and efficacy of regional citrate anticoagulation in continuous venovenous hemodialysis in the presence of liver failure: the liver citrate anticoagulation threshold (L-CAT) observational study. Crit Care. 2015, Sep, 29;19(1):349. 10.1186/s13054-015-1066-7. PMC4587580 26415638

[22] Qi W, Liu J, Li A. Regional citrate anticoagulation or heparin anticoagulation for renal replacement therapy in patients with liver failure: a systematic review and meta-analysis. Clin Appl Thromb Hemost. 2023;29:10760296231174001. 10.1177/10760296231174001. PMC10192954 37186766

[23] Zhang W, Bai M, Yu Y, Li L, Zhao L, Sun S, et al. Safety and efficacy of regional citrate anticoagulation for continuous renal replacement therapy in liver failure patients: a systematic review and meta-analysis. Crit Care. 2019, Jan, 24;2019 Jan 24(23(1):22. 10.1186/s13054-019-2317-9. PMC6345001 30678706

[24] Klingele M, Stadler T, Fliser D, Speer T, Groesdonk HV, Raddatz A. Long-term continuous renal replacement therapy and anticoagulation with citrate in critically ill patients with severe liver dysfunction. Crit Care. 2017;21(1):294. 10.1186/s13054-017-1870-3. PMC5707786 29187232

[25] Man to HH, Chit Kwan AM, Yee Leung NY, Kin Wong FC, Chan WM, Ngai CW, et al. Serum lactate levels and kinetics predict citrate accumulation in regional citrate anticoagulation for continuous renal replacement therapy. Kidney Int Rep. 2025;10(8):2852–55. 10.1016/j.ekir.2025.05.038 PMC12348182 40814594

[26] Honoré P-O, Rimmelé T, Joannes-Boyau O. Citrate anticoagulation for continuous renal replacement therapy. Intensive Care Med. 2024, Sep;50(9):1553–56. 10.1007/s00134-024-07531-3. 39037609

[27] Di Mario F, Regolisti G, Pacchiarini MC, Mordà B, Greco P, Maccari C, et al. Regional citrate anticoagulation ensures safe and effective kidney replacement therapy in metformin-associated lactic acidosis. Clin Kidney J. 2025;18(10):sfaf286. 10.1093/ckj/sfaf286 PMC12508805 41079154

[28] Boer W, van Tornout M, Solmi F, Willaert X, Schetz M, Oudemans-van Straaten H. Determinants of Total/ionized calcium in patients undergoing citrate CVVH: a retrospective observational study. J Crit Care. 2020;59:16–22. 10.1016/j.jcrc.2020.05.005. 32485438

[29] Payne RB, Little AJ, Williams RB, Milner JR. Interpretation of serum calcium in patients with abnormal serum proteins. Br Med J. 1973, Dec, 15;4(5893):643–46. 10.1136/bmj.4.5893.643. PMC1587636 4758544

[30] Khawaja I, Gopireddy NSR, Grover S, Radford G, Fraer M, Nizar JM, et al. Albumin-corrected calcium ratios for citrate accumulation increase detection and improve mortality prediction in continuous kidney replacement therapy. Crit Care. 2026, Mar, 11;30(1):111. 10.1186/s13054-026-05933-x. PMC12980849 41814403

[31] Zheng Y, Zhuang F, Zhu Q, Ma S, Xu Y, Lu J, et al. Albumin-corrected total/ionized calcium ratio is not superior to total/ionized calcium ratio as an indicator of citrate accumulation. Int J Artif Organs. 2017;40(11):602–06. 10.5301/ijao.5000621. 28708212

[32] Desgagnes N, King JA, Kline GA, Seiden-Long I, Leung AA. Use of albumin-adjusted calcium measurements in clinical practice. JAMA Netw Open. 2025;8(1):e2455251. 10.1001/jamanetworkopen.2024.55251. PMC11751745 39836424

[33] Meier-Kriesche HU, Gitomer J, Finkel K, DuBose T. Increased total to ionized calcium ratio during continuous venovenous hemodialysis with regional citrate anticoagulation. Crit Care Med. 2001;29(4):748–52. 10.1097/00003246-200104000-00010. 11373461

[34] Bai M-O, Yu Y, Zhao L, Tian X, Zhou M, Jiao J-O, et al. Regional citrate anticoagulation versus No anticoagulation for CKRT in patients with liver failure with increased bleeding risk. Clin J Am Soc Nephrol. 2024, Feb, 1;19(2):151–60. 10.2215/CJN.0000000000000351. PMC10861105 37990929

[35] Schwarzer P, Kuhn SO, Stracke S, Gründling M, Knigge S, Selleng S, et al. Discrepant post filter ionized calcium concentrations by common blood gas analyzers in CRRT using regional citrate anticoagulation. Crit Care. 2015, Sep, 8;19(1):321. 10.1186/s13054-015-1027-1. PMC4563947 26353802

[36] Chardon N, Payen D, Khadzhynov D, Rimmelé T, Bidar F. Albumin-corrected tCa/iCa ratio: are we still diagnosing citrate accumulation or predicting mortality? Crit Care. 2026;30(1). 10.1186/s13054-026-06006-9. PMC13049984 41935324

[37] Teixeira JP, Yessayan L, Tolwani A, Neyra J, Reis T. Should we change our approach to detecting citrate accumulation in patients treated with CRRT with regional citrate? Crit Care. 2026, May, 11;30(1):248. 10.1186/s13054-026-06077-8. PMC13162371 42116175

[38] Schultheiß C, Saugel B, Phillip V, Thies P, Noe S, Mayr U, et al. Continuous venovenous hemodialysis with regional citrate anticoagulation in patients with liver failure: a prospective observational study. Crit Care. 2012, Aug, 22;16(1466-609X (Electronic)):R162. 10.1186/cc11485. PMC3580752 22913794

